# Metabolic syndrome and mean platelet volume variation in patients with chest pain and negative cardiac enzymes

**Published:** 2013-06-25

**Authors:** AC Nechita, C Delcea, V Enache, RL Ploesteanu, C Cazacu, AM Andronescu, AM Stroi, CS Stamate

**Affiliations:** * 1st Internal Medicine and Cardiology Department, "Sfantul Pantelimon" Clinical Emergency Hospital Bucharest; ** "Carol Davila" University of Medicine and Pharmacy Bucharest; *** Neurology Department, Colentina Clinical Hospital

**Keywords:** mean platelet volume, MPV, metabolic syndrome, unstable angina

## Abstract

Introduction. The mean platelet volume (MPV) is an easily measurable parameter directly correlated with platelet aggregation function, proven to be increased in acute coronary syndromes, but also in the presence of cardiovascular risk factors such as the metabolic syndrome, dyslipidemia, diabetes mellitus, arterial hypertension.

Objective. This study intended to assess the role of the metabolic syndrome in MPV variation in patients presenting with chest pain.

Materials and Methods. We retrospectively analyzed data from 122 patients with chest pain and negative cardiac enzymes admitted consecutively to our clinic from September 1st 2011 to January 30th 2012. Our group included 27 (22.13%) patients with stable angina (SA), 74 (60.65%) patients with unstable angina (UA) and 21 (17.22%) patients with non-coronary chest pain.

Results. Patients with UA had a higher mean value of the MPV 9.31 ± 1.19 fL compared to patients with SA 8.72 ± 1.14 fL (p=0.0279) and patients with non-coronary chest pain 8.85 ± 0.90 L (p=0.0908). All the patients with metabolic syndrome had increased MPVs, regardless of the etiology of chest pain. Patients with non-coronary chest pain presented significantly higher MPVs if associated with metabolic syndrome or arterial hypertension.

Conclusions. Patients with cardiovascular risk factors, especially complex ones like the metabolic syndrome had an increased MPV, as did the patients with UA whether or not associated with the risk factors. In patients without such comorbidities, the MPV could be useful in distinguishing unstable angina from non-coronary chest pain.

## Introduction

Platelet aggregation function is directly correlated with the mean platelet volume (MPV), a larger MPV being an indicative of a more active platelet population [**1**]. Not only an independent risk factor for myocardial infarction, but an increased MPV has also been associated with the severity of unstable angina [**2,3**]. This easily measurable parameter was also proven to be one of the predictors of prognostic in patients with acute coronary syndromes, an increased MPV suggesting worse outcomes [**4,5**]. This hypothesis is also supported by the observation that an increased MPV contributes to the prethrombotic state in this type of cardiac pathology [**6**].

 MPV was found to be elevated not only in patients with coronary artery disease, but also in patients with clinically manifest cardiovascular risk factors. The presence of diabetes mellitus was strongly and independently associated with an increased MPV [**7**] and more so, poor glycemic control induced an increased platelet activity in patients previously diagnosed with this pathology [**8**]. MPV was positively correlated to the arterial blood pressure, hypertension leading to an increased MPV [9,10], MPV values being associated with the severity of subclinical target organ damage [**11**]. Meanwhile, the presence of the metabolic syndrome and its relation to the variation of MPV is still controversial, with evidence suggesting an association between the two entities [**12,13**] as well as evidence against it [**7,15,17**].

### Objective

The aim of our study was to determine to what extent the metabolic syndrome and its components influenced the mean platelet volume variation in patients presenting with chest pain, in order for it to be used in the differential diagnosis of this very common, sometimes misleading symptom.

## Materials and Methods

We conducted a retrospective study of 122 patients with chest pain and negative cardiac enzymes admitted consecutively to the Cardiology Department of “Sfantul Pantelimon" Emergency Hospital in Bucharest, Romania, from September 1st 2011 to January 30th 2012. Inclusion criteria consisted of chief complaint of chest pain suggestive of angina and troponin I, troponin T, CK, CK-MB within normal limits, without EKG changes suggestive of an acute ST segment elevation myocardial infarction. Exclusion criteria comprised any complete blood count abnormality such as anemia, thrombocytopenia, active external or internal bleeding, suspected internal bleeding, elevated serum levels of cardiac necrosis markers, ST segment elevation myocardial infarction criteria present on the EKG.

 Our group consisted of 27 (22.13%) patients with stable angina (SA), 74 (60.65%) patients with unstable angina (UA) and 21 (17.22%) patients with non-coronary chest pain. 65 (53.28%) were females and 57 (46.72%) males, with a mean age of 62.13 ± 15.18 years old.

### Statistical analysis

Statistical analysis Data was processed by using SPSS 16.0 and Epi.Info 7. Parametric variables were expressed as mean value ± standard deviation. Bartlett's test was used to assess homogeneity of distributions. For normally distributed data, the Anova test was computed and for the non-normally distributed data, the Mann-Whitney U test. A p value less than 0.05 was considered statistically significant.

## Results

A total of 122 patients were analyzed, after being divided into three subgroups according to the etiology of chest pain. Mean age of the entire lot was of 62.13 ± 15.18 years old. Of these, patients complaining of chest pain who proved to be non-coronary in origin were significantly younger, with a mean age of 55.38 ± 16.95 years old, age range 46 to 84 years old compared to patients with UA who had a mean age of 63.68 ± 13.82 years old, age range 53 to 89 years old (p=0.0231), as well as patients with SA, who had a mean age of 63.11 ± 16.44 years old, age range 54 to 88 years old (p=0.0789). 

 Male to female ratio was similar in all patients, with a slight female predominance.

 Regarding vital signs on arrival, systolic blood pressure (SBP) ranged from 90 to 235 mmHg with a mean value of 145.71 ± 25.01 mmHg, and diastolic blood pressure (DBP) from 50 to 120 mmHg, with a mean value of 78.22 ± 12.09 mmHg. Values were similar in all three subgroups, with mean SBP and DBP without statistically significant variation. The same concordance was observed regarding heart rate (HR), ranging from 46 to 148 beats per minute (bpm) with a mean value of 78.31 ± 19.46 bpm, similar in all subgroups, regardless of the cause of chest pain (**Table 1**).

**Table 1:** Gender distribution, mean age and vital signs according to the etiology of chest pain

	Unstable Angina	Stable Angina	Non-Coronary Chest Pain
	N = 74 pts	N = 27 pts	N = 21 pts
Male Sex	35 (47.29%)	13 (48.14%)	9 (42.85%)
Age (years)	63.68 ± 13.82	63.11 ± 16.44	55.38 ± 16.95*
SBP (mmHg)	145.69 ± 28.61	145.40 ± 16.90	146.19 ± 21.08
DBP (mmHg)	78.23 ± 13.07	78.26 ± 9.37	78.14 ± 12.14
HR (bpm)	79.44 ± 19.22	76.88 ± 19.10	76.28 ± 21.34
* p value < 0.05

Prevalence of cardiovascular risk factors was assessed, focusing on the metabolic syndrome and its components. The metabolic syndrome was discovered in 49.18% of all patients, with similar frequency in all subgroups: 47.29% in patients with UA, 51.85% in patients with SA and 52.38% in patients with non-coronary chest pain. Similar values were discovered regarding the presence of central obesity. The most common risk factors were hypertension with a general preponderance of 80.33% and dyslipidemia with a frequency of 68.85% in the entire group, similar values for patients with angina and slightly less common in patients without coronary artery pathology (**Table 2**). Fewer patients were diagnosed with diabetes mellitus or altered fasting plasma glucose levels, this risk factor being encountered in only 22.13% of the patients studied. 

**Table 2:** Risk factor prevalence among patients with different etiologies of chest pain

	Unstable Angina	Stable Angina	Non-Coronary Chest Pain
	N = 74 pts	N = 27 pts	N = 21 pts
Metabolic Syndrome	35 (47.29%)	14 (51.85%)	11 (52.38%)
Central Obesity	37 (50.00%)	14 (51.85%)	13 (61.90%)
Altered fasting glucose levels or diabetes mellitus	21 (28.38%)	4 (14.81%)	6 (28.57%)
Dyslipidemia	53 (71.62%)	16 (59.26%)	15 (71.43%)
Arterial Hypertension	60 (81.08%)	22 (81.48%)	16 (76.19%)

 Mean platelet volume (MPV) variation was assessed in all three subgroups. Patients with UA were proved to have a higher mean value of the MPV 9.31 ± 1.19 (6.94 - 12.60) fL compared to the patients with SA with a mean MPV of 8.72 ± 1.14 (6.81 - 11.80) fL (p=0.0279) and patients with non-coronary chest pain with a mean MPV of 8.85 ± 0.90 (7.44 - 10.90) fL (p=0.0908). Platelet number was similar in all three subgroups, without a significant variation. Patients with UA had a mean platelet number of 256270.27 ± 62079.33/uL, patients with SA 261777.77 ± 62541.88/uL and patients with non-coronary chest pain 266857.14 ± 70053.04/uL.

**Graph 1:**
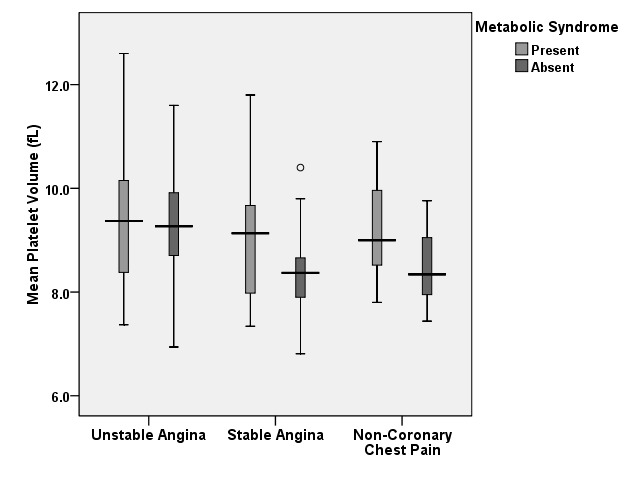
Mean platelet volume variation in patients with/ without metabolic syndrome

We then analyzed each subgroup from the perspective of the risk factors. In patients with UA, MPV was found to vary slightly concerning the diagnosis of metabolic syndrome. The mean values remained elevated as compared to the other subgroups in both subjects with UA and metabolic syndrome (9.41 ± 1.36 fL) and patients with UA without metabolic syndrome (9.22 ± 1.04 fL). In patients with SA, the presence of the metabolic syndrome was associated with a slight increase in mean MPV 9.03 ± 1.22 fL in comparison to 8.39 ± 0.99 fL. The most important difference was found in patients with non-coronary chest pain. Their mean MPV was of 8.44 ± 0.68 fL if the metabolic syndrome was absent and 9.22 ± 0.93 fL in its presence (p=0.0439). Comparing the mean MPVs of patients with the metabolic syndrome, we observed that in this case, there are no significant variations between the different etiologies of chest pain. In patients without the metabolic syndrome, patients with UA maintain the highest mean MPV of 9.22 ± 1.04 fL compared to the patients with non-coronary chest pain with a mean MPV of 8.44 ± 0.68 fL (p=0.0305) and to those with SA with a mean MPV of 8.39 ± 0.99 fL (p=0.0152) (**Graph 1**).

**Graph 2:**
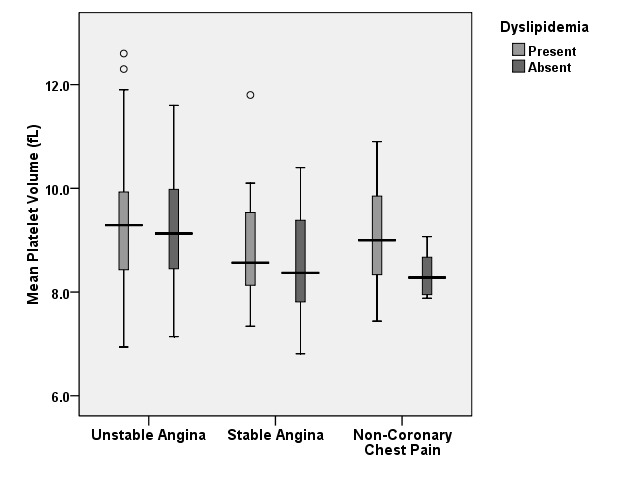
Mean platelet volume variation in patients with/ without dyslipidemia

Same variations were found in patients with/without dyslipidemia (**Graph 2**). All dyslipidemia patients, regardless of the etiology of the chest pain, had similar MPVs: 9.33 ± 1.23 fL for patients with UA, 8.86 ± 1.11 fL for patients with SA and 9.05 ± 0.96 fL. Patients without dyslipidemia maintained the proportions discovered in the initial subgroups with the highest mean MPV in patients with UA 9.26 ± 1.12 fL vs 8.52 ± 1.20 fL in patients with SA (p=0.0925) and 8.35 ± 0.46 fL in patients with non-coronary chest pain (p=0.0507). No statistically significant difference was found within the subgroups in accordance with the presence of dyslipidemia, even though an increasing trend was observed in patients with this risk factor.

**Graph 3:**
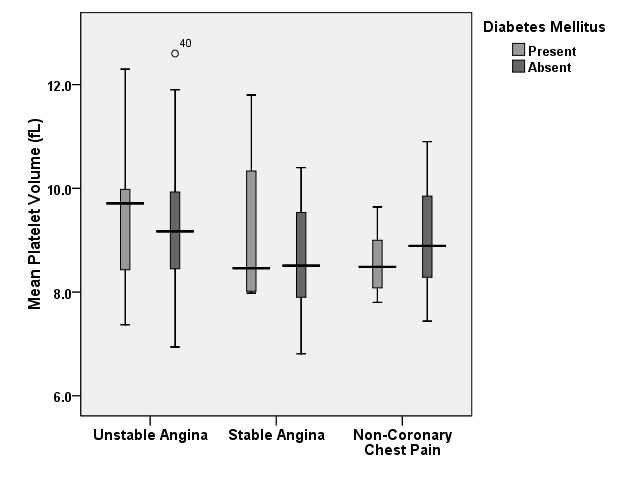
Mean platelet volume variation in patients with/ without diabetes mellitus

 We also analyzed MPV variation influenced b the presence or absence of diabetes mellitus (**Graph 3**). Small differences were found in patients without UA, with an MPV slightly higher 9.17 ± 1.79 fL vs 8.64 ± 1.03 fL in patients with SA and 8.58 ± 0.67 fL vs 8.96 ± 0.97 fL in patients with non-coronary chest pain. Patients with UA had very similar, increased mean MPVs in both subgroups: 9.48 ± 1.24 fL in those with diabetes and 9.24 ± 1.18 fL in those without.

**Graph 4:**
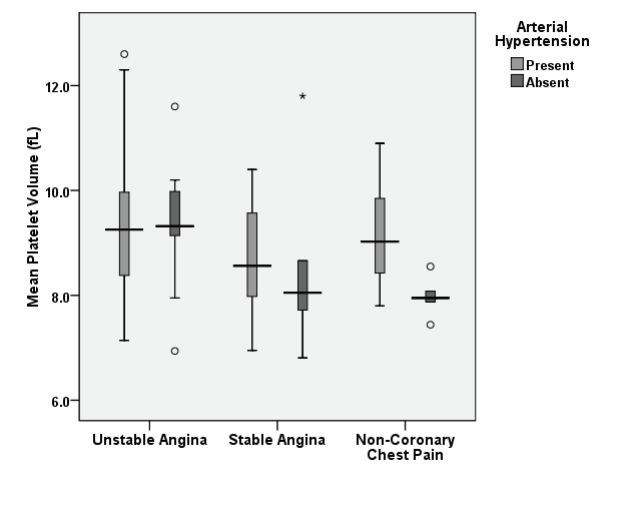
Mean platelet volume variation in patients with/ without arterial hypertension

One last risk factor that was found to influence MPV variation was the arterial hypertension (HTN) (Graph 4). The highest impact was found in patients with non-coronary chest pain whose mean MPV was 7.98 ± 0.39 fL for the subjects with normal blood pressure compared to 9.12 ± 0.83 fL for those with elevated blood pressure. No statistically significant difference was found in subgroups of patients with SA with/without HTN, whose mean MPV was of 8.75 ± 0.96 fL vs. 8.60 ± 1.90 fL. A similar variation was determined for subjects with UA with/without HTN :9.30 ± 1.23 fL vs. 9.34 ± 1.18 fL. The initial relationship between patients with UA and those with non-coronary chest pain remains valid in those without HTN, as the mean MPV is significantly higher in the first subgroup (p=0.0149).

## Discussion

Known to be associated with acute coronary syndromes, MPV is an indicative of platelet activation and increased aggregation. Previous studies have shown, however, that MPV is enlarged not only in patients with acute cardiac ischemia [**3**], but also in stable patients with metabolic syndrome [**12,13**], hypertension [**9,10**], dyslipidemia [**16-18**], diabetes mellitus [**7,19**]. 

 Our study compared groups of patients presenting with chest pain and negative cardiac enzymes to assess to what extent the differences in MPV are preserved when patients are also diagnosed with one or more of the components of the metabolic syndrome known to increase MPV. 

 At a first glance, as previous studies have shown, patients with UA have statistically significant higher values of the MPV when compared to patients with SA or non-coronary chest pain. However, when we analyzed subgroups of patients with and without risk factors, we observed that approximately all patients with risk factors have an increased MPV compared to the individuals without risk factors. When analyzing the subgroup diagnosed with unstable angina, the mean value of the MPV was higher than the subgroups with stable angina or non-coronary chest pain, regardless of the presence or absence of the risk factors, thus underlining the independently increased values.

 Most variation within a subgroup with the same etiology for the chest pain was found in patients with non-coronary chest pain in accordance to the presence of risk factors that increased the mean values of the MPV significantly. Considering these patients free of cardiac disease burden, we are able to observe in this subgroup the dynamic of the MPV in strict relationship to the presence of risk factors. The highest impact was observed for the metabolic syndrome as well as for hypertension, these co morbidities leading to a significant increase in the value of MPV.

 In patients with stable angina, an increasing tendency was observed in all subgroups analyzed from the point of view of many risk factors, however with borderline or trending p values for statistical significance. The metabolic syndrome played the most important role in increasing the MPV in these patients, as compared with the arterial hypertension, diabetes mellitus or dyslipidemia.

## Conclusion

Patients with cardiovascular risk factors, especially complex ones such as the metabolic syndrome have increased values of the MPV, regardless of the etiology of chest pain thus making this parameter unlikely to be useful in the differential diagnosis in these circumstances.

 In the absence of cardiovascular risk factors, as in the general population, patients with UA have the highest mean values for the MPV, statistically significant higher than patients with SA or non-coronary chest pain, who have similar values. 

 The MPV could serve as an additional factor to be considered in the differential diagnosis of patients with chest pain with negative cardiac enzymes, however, only in the absence of the major cardiovascular risk factors such as the metabolic syndrome, dyslipidemia, diabetes mellitus or arterial hypertension.
